# The EBNA-2 N-Terminal Transactivation Domain Folds into a Dimeric Structure Required for Target Gene Activation

**DOI:** 10.1371/journal.ppat.1004910

**Published:** 2015-05-29

**Authors:** Anders Friberg, Sybille Thumann, Janosch Hennig, Peijian Zou, Elfriede Nössner, Paul D. Ling, Michael Sattler, Bettina Kempkes

**Affiliations:** 1 Institute of Structural Biology, Helmholtz Zentrum München, National Research Center for Environmental Health, Neuherberg, Germany; 2 Center for Integrated Protein Science Munich at Chair Biomolecular NMR Spectroscopy, Department Chemie, Technische Universität München, Garching, Germany; 3 Department of Gene Vectors, Hematologikum, Helmholtz Zentrum München, National Research Center for Environmental Health, München, Germany; 4 Industrial Enzymes National Engineering Laboratory, Tianjin Institute of Industrial Biotechnology, Chinese Academy of Sciences, Tianjin, China; 5 Institute of Molecular Immunology, Hematologikum, Helmholtz Zentrum München, National Research Center for Environmental Health, München, Germany; 6 Department of Molecular Virology & Microbiology, Baylor College of Medicine, Houston, Texas, United States of America; Wistar Institute, UNITED STATES

## Abstract

Epstein-Barr virus (EBV) is a γ-herpesvirus that may cause infectious mononucleosis in young adults. In addition, epidemiological and molecular evidence links EBV to the pathogenesis of lymphoid and epithelial malignancies. EBV has the unique ability to transform resting B cells into permanently proliferating, latently infected lymphoblastoid cell lines. Epstein-Barr virus nuclear antigen 2 (EBNA-2) is a key regulator of viral and cellular gene expression for this transformation process. The N-terminal region of EBNA-2 comprising residues 1-58 appears to mediate multiple molecular functions including self-association and transactivation. However, it remains to be determined if the N-terminus of EBNA-2 directly provides these functions or if these activities merely depend on the dimerization involving the N-terminal domain. To address this issue, we determined the three-dimensional structure of the EBNA-2 N-terminal dimerization (END) domain by heteronuclear NMR-spectroscopy. The END domain monomer comprises a small fold of four β-strands and an α-helix which form a parallel dimer by interaction of two β-strands from each protomer. A structure-guided mutational analysis showed that hydrophobic residues in the dimer interface are required for self-association *in vitro*. Importantly, these interface mutants also displayed severely impaired self-association and transactivation *in vivo*. Moreover, mutations of solvent-exposed residues or deletion of the α-helix do not impair dimerization but strongly affect the functional activity, suggesting that the EBNA-2 dimer presents a surface that mediates functionally important intra- and/or intermolecular interactions. Our study shows that the END domain is a novel dimerization fold that is essential for functional activity. Since this specific fold is a unique feature of EBNA-2 it might provide a novel target for anti-viral therapeutics.

## Author Summary

Epstein-Barr virus is an oncogenic γ-herpesvirus that may cause infectious mononucleosis in young adults and fatal lymphoproliferative disorders in immunocompromised patients and is associated with the pathogenesis of Burkitt's lymphoma, nasopharyngeal and gastric carcinoma. Epstein-Barr virus nuclear antigen 2 (EBNA-2) is a key regulator of viral and cellular gene expression which initiates and maintains a specific transcription program that promotes proliferation and differentiation of the infected B cell. EBNA-2 is a transcriptional activator that is recruited to DNA by cellular adaptor proteins, carries two transactivation domains, and has the capacity to form dimers or multimers. This study provides the first three-dimensional structure of the EBNA-2 N-terminal Dimerization (END) domain. Two END domain monomers, each consisting of four β-strands and a single α-helix, assemble into a dimer by interaction of two β-strands from each monomer in a parallel fashion. The dimer surface exposes residues that are critical for transactivation of target genes by EBNA-2. The dimeric fold of the EBNA-2 END domain has not been observed for any cellular protein and thus could provide a novel target for anti-viral therapeutics.

## Introduction

Epstein-Barr virus (EBV) is a γ-herpesvirus that establishes a lifelong asymptomatic infection in the majority of human adults. EBV infection or reactivation can cause significant morbidity and mortality in immunocompromised transplant recipients of allogeneic hematopoietic stem cells or solid organs [1, 2]. EBV has the unique ability to transform resting human B cells into permanently proliferating latently infected lymphoblastoid cell lines. This process is controlled by the concerted action of six latent EBV nuclear antigens (EBNAs) and three latent membrane proteins (LMPs), which mimic cellular functions required for B cell proliferation and differentiation. EBNA-2 is a key viral factor in the initiation of the transformation process. The protein controls a specific transcription program that is associated with proliferation of the infected B cells and that closely resembles transcript patterns of EBV infected B cells described in post-transplant lymphoproliferative disorders (PTLD) of immunosuppressed patients [3]. Thus, EBNA2 could potentially serve as a target to develop therapeutic strategies which interfere with the proliferation of EBV positive PTLD originating from B cells. Structural information on EBNA2 could guide the development of new antivirals in the future.

EBV belongs to the genus of lymphocryptoviruses (LCV) and is the only LCV species that infects humans. Mainly based on the sequence diversity of the EBNA-2 alleles EBV can be categorized in two individual strains called type 1 and 2. Type 1 and 2 EBV strains differ in their capacity to immortalize primary B cells [4, 5] which is predominantly determined by sequence variation in the C-terminus of EBNA-2 [6, 7]. Most researchers in the field use the laboratory EBV strain B95-8 (type 1) which encodes a 487 amino acid EBNA-2 protein [8, 9]. Lymphocryptoviruses have also been isolated from baboon and macaque. While the EBNA-2 orthologs of baboon and macaque LCV show significant amino acid similarity with EBNA-2 encoded by the B95-8 strain [10, 11], similarity with the positional EBNA-2 homolog of marmoset LCV is below 20% (reviewed in [12]).

**Fig 1:**
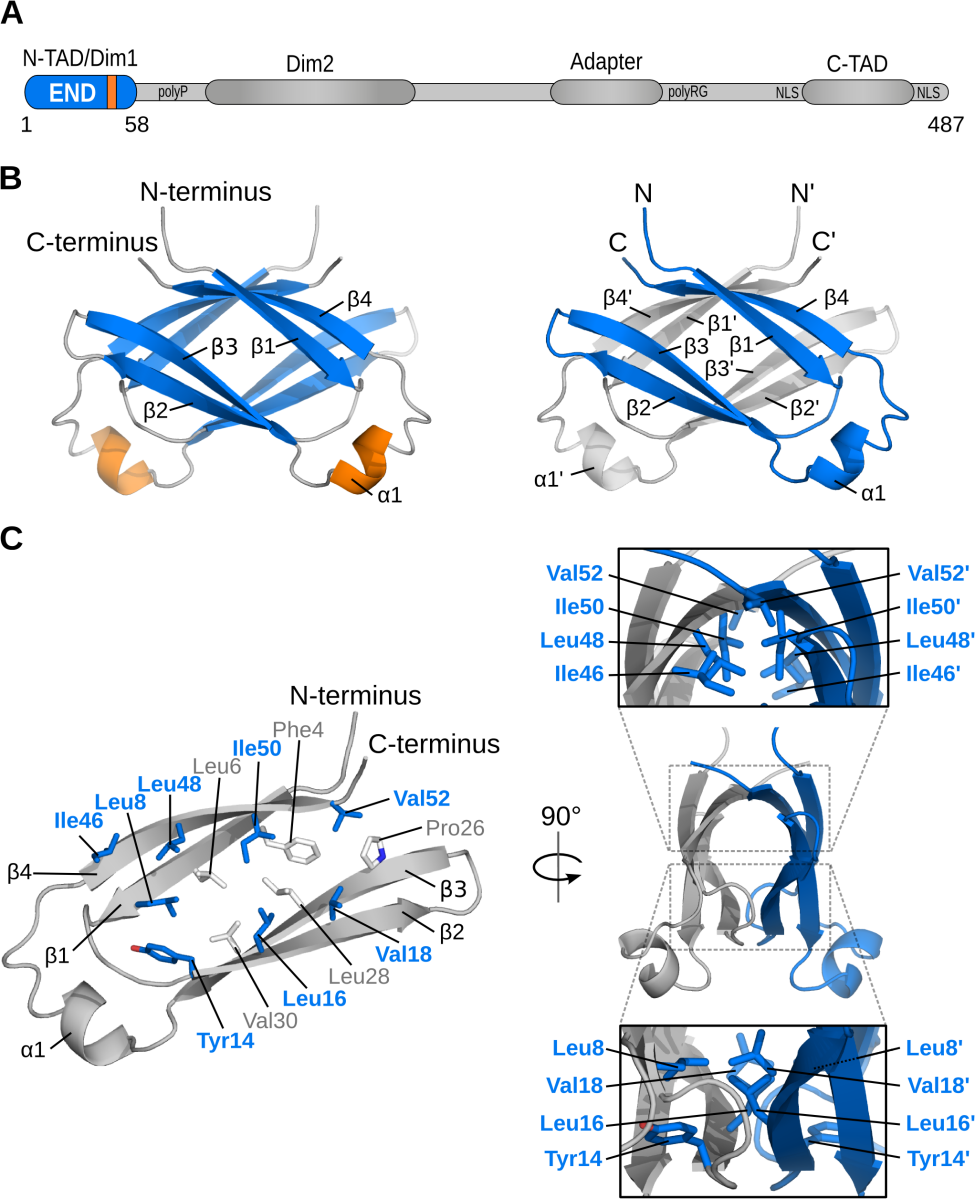
Structure of the EBNA-2 N-terminal dimerization (END) domain. Schematic representation of important features of the EBNA-2 protein: two dimerization motifs (Dim1/Dim2), N-terminal and C-terminal transactivation domains (N-TAD, C-TAD), repetitive primary sequence motifs like the poly-proline (polyP) and the poly arginine-glycine (polyRG) stretch, the nuclear localization signals (NLS),and the adapter region of EBNA-2, which interacts with CBF1/CSL, are illustrated. (B) NMR solution structure of the END (EBNA-2 N-terminal Dimerization) domain. Left: β-strands are shown in blue, helices in orange, and loops in gray. Right: Monomers highlighted in gray and blue. (C) Dimerization of monomers is stabilized by hydrophobic interactions. The inside of each monomer is lined with numerous hydrophobic residues (left; sticks). A subset of these residues is located at the dimer interface (blue/bold labels). Panels (right) show side views of the END domain and highlight the interface residues of each monomer.

The transactivator EBNA-2 does not bind to DNA directly but uses cellular DNA binding proteins like CBF1/CSL as adapters to gain access to enhancer and promoter sites in the viral and cellular genome (reviewed in [13]). Two transactivation domains have been mapped within the primary structure of the EBNA-2 protein by tethering EBNA-2 fragments fused to the yeast GAL4 DNA binding domain to GAL4 dependent reporter genes (Fig 1A). The C-terminal acidic transactivation (C-TAD, aa 448–471) domain can recruit components of the basic transcriptional machinery like TFIIE via p100, TFIIB, TAF40, to the TFB1/p62 subunit of the TFIIH complex, RBP70 [14–18] and chromatin modifiers like p300/ CBP and PCAF [19] and might directly bind to the viral co-activator EBNA-LP [20]. The EBNA-2 C-TAD is intrinsically unstructured as shown by NMR. However, the C-TAD forms a 9-residue amphipathic α-helix when bound to the pleckstrin homology (PH) domain of the yeast homolog of fragments of the TFB1/p62 subunit of the TFIIH complex. Three hydrophobic residues (Trp458, Ile461, and Phe462) of this α-helix directly contact the TFB1 PH domain. The same EBNA-2 residues are critical for the interaction with CBP/p300 [21].

A second transactivation domain has been mapped to the N-terminus (N-TAD, aa 1–58) of the EBNA-2 protein [22]. The molecular mechanism by which this second EBNA-2 transactivation domain acts has not yet been elucidated. Like the C-TAD its activity can be enhanced by EBNA-LP although it does not bind directly to EBNA-LP [22–24]. When GAL4 DNA binding domain fusion proteins of the N- or C-TAD are compared directly, they score equally well in transient transactivation assays [22]. Deletion of the N-terminus causes a severe loss of activity, while deletion of the C-TAD completely abolishes transactivation of target genes indicating that the function of the two transactivation domains are neither equivalent nor redundant [15, 25]. The relevance of the N-terminus of EBNA-2 for the growth transformation process has been studied in two independent cellular systems. The results of both studies suggested that the N-terminus of EBNA-2 is of major importance for the transformation efficiency of the virus and the survival of EBV infected B cells [24, 26].

Two N-terminal regions separated by a poly-proline stretch have been proposed to mediate homotypic self-association of EBNA-2. The first, consisting of amino acid 1–58 coincides with the N-terminal transactivation domain [22, 23]. A second self-associating region is composed of amino acid 97–121 [23]. An additional self-associating domain has been mapped to a non-conserved region which is flanked by the second dimerization and the adapter region [27].

The N-terminal region of EBNA-2 comprising residues 1–58 appears to mediate multiple molecular functions including self-association, transactivation and functional cooperation with EBNA-LP. Similar functions have also been assigned to other parts of the protein. So far it is unknown if the N-terminus of EBNA-2 directly provides all these functions or if these activities merely depend on the dimerization involving the N-terminal domain. Thus, the molecular basis and functional importance of the dimerization regions are poorly understood since three-dimensional structural data for the entire EBNA-2 protein have not been reported.

Here, we present the three-dimensional structure of the EBNA-2 N-terminus which forms a compact parallel homodimer that is stabilized by a hydrophobic interface between the two monomers. The dimer interface involves two β-strands of each protomer that pack against each other in an anti-parallel manner. Based on this structural information we generated site-directed mutants which target either the hydrophobic dimer interface or solvent-exposed residues. We show that interface mutations abolish self-association of EBNA-2 and severely impair its transactivation function. Notably, surface mutants do not impair self-association. However, specific point mutations or deletion of a protruding α-helix on the surface of the END domain cause a major loss of biological activity. These data suggest that the EBNA-2 dimer provides a surface that is critical for its transactivation function.

## Results

### The N-terminal domain of EBNA-2 is a novel structural dimerization motif

Structure predictions for the full-length EBNA-2 amino acid sequence suggest that this viral protein does not form a globular three-dimensional fold, consistent with the presence of extended poly-proline or poly-glycine-arginine regions, and with a total proline content of 28%. The EBNA-2 protein thus appears to comprise intrinsically unstructured regions, which require interaction partners for proper folding. However, in silico analysis of the primary structure using PSIPRED [28], predicts that the N-terminal region comprises β-strands and thus might represent a small globular domain (Fig A in S1 Text).

To characterize biochemical and structural details of this region of EBNA-2, an N-terminal fragment comprising residues 1–58 was expressed in *E*.*coli* and purified with or without Z-tag under native conditions. The oligomerization status of the recombinant proteins was analyzed by analytical size exclusion chromatography (SEC) and static light scattering (SLS) (Table 1).The EBNA-2 N-terminal fragment lacking a Z-tag forms a single molecular species with a molecular mass of 13.1 kDa as expected for a dimer (2x6.7 kDa). Similarly, the EBNA-2 Z-tag fusion protein eluted as a single peak with a molecular mass of 46.3 kDa close to the theoretical molecular mass of a dimer (2x23.4 kDa).

**Table 1:** Dimerization analysis of wild-type and mutant END domains by SEC/SLS and NMR.

Construct	SLS, without tag ^A^	SLS, with Z-tag ^A^	2D NMR ^B^
Wild-type	Dimer	Dimer	Dimer
*Interface mutants*			
L16A	Monomer	Monomer	Monomer/Dimer ^C^
L16D	Aggregation	Aggregation	ND
I50A	Aggregation	Aggregation	ND
I50D	Aggregation	Aggregation	ND
*Surface mutants*			
H15A	Dimer	Dimer	Dimer
F51A	Dimer/ (Aggregation)	Dimer/ (Aggregation)	Dimer/Aggregation
Δα1	Dimer/ (Aggregation)	Dimer/ (Aggregation)	Dimer/Aggregation

ND—Not determined. Protein sample not stable and/or not suitable for NMR analysis.

^A^ Molecular weights were calculated from refractive index (RI) and right angle light scattering (RALS) data (Fig D in S1 Text).

^B^ For NMR, proteins without a Z-tag were analyzed.

^C^ 2D ^1^H,^15^N-HSQC spectrum indicates the presence of two populations, interpreted as an equilibrium between a folded dimer and the unfolded monomer of the END domain (Fig D in S1 Text).

**Fig 2:**
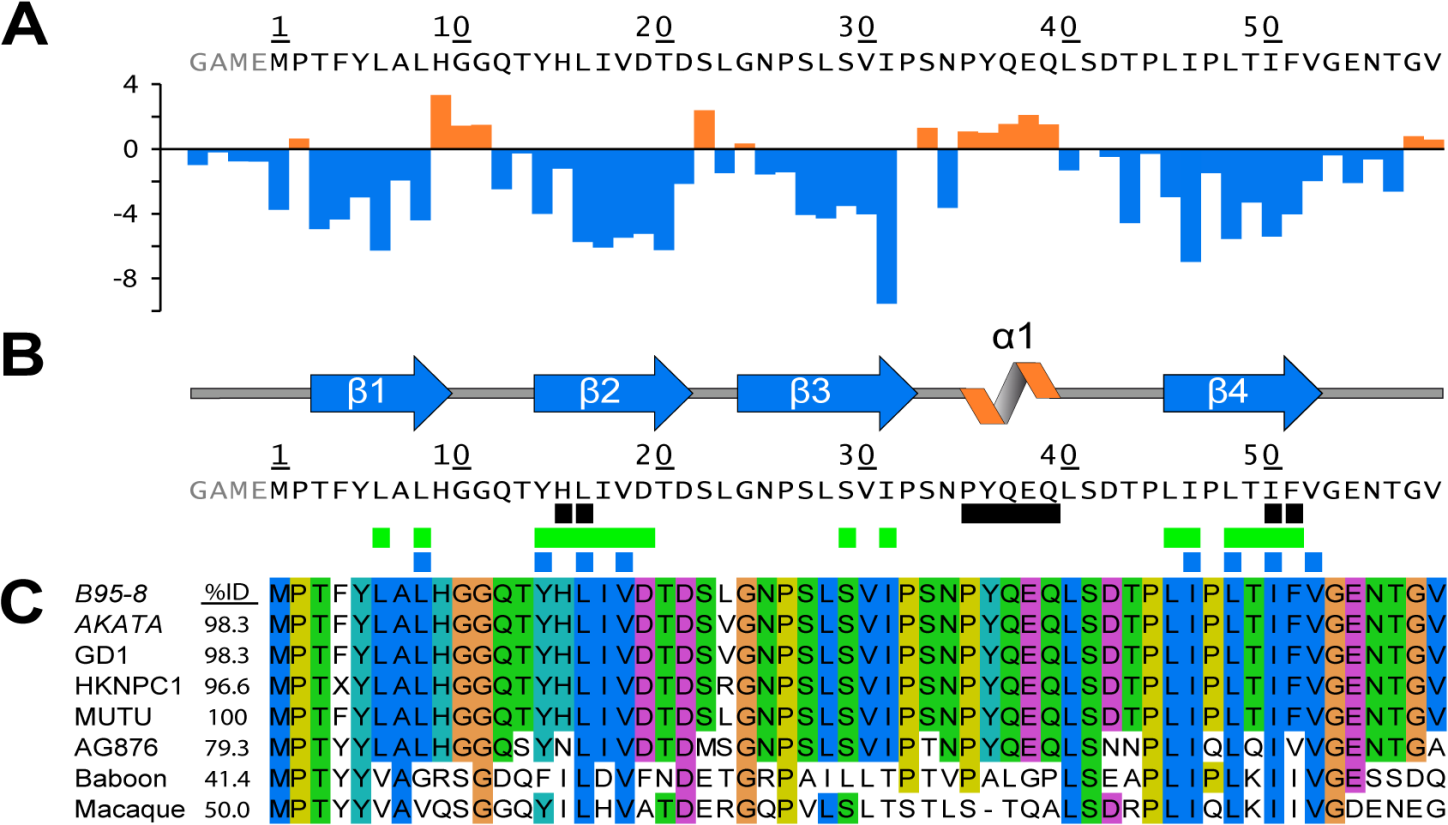
Secondary structure topology of the END domain and sequence alignment. (A) Calculated secondary chemical shifts, Δδ(^13^Cα-^13^Cβ), of the END domain. Positive (orange) and negative (blue) values indicate propensity for α-helical and β-strand conformation, respectively. (B) Secondary structure elements of the END domain based on the NMR structure. Black rectangles indicate residues included in our mutational analysis (for details see Fig 3A). Green rectangles mark backbone amides protected from solvent exchange in hydrogen-deuterium exchange experiments (Fig D in S1 Text). Blue rectangles show the hydrophobic core residues of the END domain forming the interface between the two dimers (Fig 1C). (C) Multiple sequence alignment of potential EBNA-2 END domains in human and related monkey viruses. The construct of this study was based on type 1 EBV strain B95-8 (P12978). The B95-8 sequence was aligned to several type 1 EBV strains (AKATA: AFY97831.1; GD1: Q3KSV2.1; HKNPC1: AFJ06836.1; MUTU: AFY97916.1), the type 2 EBV strain AG876 (YP_001129441.1), and to the LCV strains from baboon (AAA79034.1) and macaque (YP_067943.1). A residue is conserved and colored if the sequence identity over all displayed sequences is higher than 60%. The color code for the amino acid residues is as follows: hydrophobic (blue: M, F, L, I, V, A), small polar (green: T, Q, S, N), aromatic polar (cyan: Y, H), negatively charged (magenta: D, E), glycine (orange), proline (yellow).

We next determined the three-dimensional structure of this N-terminal fragment by heteronuclear nuclear magnetic resonance (NMR) spectroscopy. The solution structure of the N-terminal domain is well-defined by the NMR data and based on more than 1250 nuclear Overhauser effect (NOE)-derived distance restraints per monomer and 205 inter-monomer NOEs (Table 2). The structure reveals a parallel homodimeric arrangement of monomers each comprising four β-strands (β1-β4) and a short exposed α-helix (α1) remote from the dimer interface (Fig 1B and 1C). The central portion of the dimer is assembled by two curved anti-parallel β-sheets with an anti-parallel arrangement of β1-β4 with β4’-β1’ and β3-β2 with β2’-β3’ (un/primed secondary structures refer to the individual monomers). The dimer interface is constituted by anti-parallel interactions of β4-β4’ and β2-β2’, respectively (Fig 1B and 1C, right panel). The secondary structure observed in the structure is consistent with NMR secondary chemical shifts (Fig 2A). Structural similarity searches in the Protein Data Bank (PDB) using DALI and PDBeFold did not identify any structures with a similar fold (see Experimental Methods for details). Thus, the N-terminal domain of EBNA-2 represents a novel dimerization fold, which we propose to name “END” (EBNA-2 N-terminal Dimerization) domain.

**Table 2:** Structural statistics.

**Experimental restraints**	
Distance restraints ^A^	2522
Intra-residue	456
Inter-residue	
Short-range (|i-j| = 1)	702
Medium-range (1< |i-j| <5)	246
Long-range (|i-j| >5)	708
Inter-monomer	410 (2x205)
Dihedral restraints (ϕ/ψ)	176 (2x88)
**Structural quality**	
Coordinate precision (Å) ^B^ *	
N, Cα, C'	0.35 ± 0.08
Heavy atoms	0.66 ± 0.05
Restraint RMSD ^C^	
Distance restraints (Å)	0.015 ± 0.003
Dihedral restraints (°)	0.808 ± 0.102
Deviation from idealized geometry ^D^	
Bond lengths (Å)	0.008
Bond angles (°)	1.0
Ramachandran plot (%) ^E^ *	
Preferred regions	93.4
Allowed regions	6.6
Generously allowed regions	0
Disallowed regions	0
WhatIf analysis ^F^ *	
1^st^ generation packing	3.218 ± 1.105
2^nd^ generation packing	7.898 ± 2.540
Ramachandran plot appearance	1.245 ± 0.578
Chi-1/Chi-2 rotamer normality	-3.492 ± 0.454
Backbone conformation	2.384 ± 0.614

^A^ 2412NOE cross peaks out of 2727 were assigned by CYANA. All numbers are given for the symmetric dimer.

^B^ RMSD of the backbone coordinates to the mean structure.

^C^ Analyzed by iCING. No distance/dihedral angle restraint was violated by more than 0.3 Å/5°, respectively, in any of the models.

^D^ PDB validation and deposition server (ADIT).

^E^ With Procheck.

^F^ Structure Z-scores, a positive number is better than average.

* For residue 5–57 of the expression construct (RMSD < 2 Å).

The END domain is highly stable with a melting point of approximately 70°C (determined by thermal denaturation [29]). A strong interaction between the monomers is also consistent with a large buried surface area (1165 Å^2^, corresponding to one quarter of the total surface area per monomer) [30]. NMR relaxation data show that the folded region of the END domain between β1-β4 is highly rigid, while C-terminal residues (beyond Asn55) are flexible and exhibit internal dynamics at sub-nanosecond timescales (Fig A in S1 Text).

The END homodimer is stabilized by the formation of a hydrophobic core involving numerous residues from each monomer (Fig 1C). While some of these residues mainly stabilize interactions within each monomer, the dimer interface is formed by hydrophobic interactions of the side chains of Leu8, Tyr14, Leu16, Val18, Ile46, Leu48, Ile50, and Val52. Also, stacking of the solvent exposed side chains of His15 and Phe51 from both monomers contributes to the dimer interface. In addition to the hydrophobic interactions, hydrogen bonds between the peptide backbone of β2 and β2’, as well as β4 and β4’ are formed. These backbone interactions are supported by NMR-detected hydrogen-to-deuterium (H/D) exchange measurements, which indicate that most of the backbone amide protons that participate in intra-monomer or inter-monomer hydrogen bonds are protected against solvent exchange (Fig 2B and Fig B in S1 Text).

Taken together our structural and biophysical data shows that the recombinant wild-type END domain folds independently into a very stable dimer. Thus, we expect that the determined protein structure indicates a native assembly of the EBNA-2 protein and decided to further characterize and validate the dimer structure and its function using site-directed mutational analysis in vitro and in vivo.

### Mutational analysis of the END domain in vitro

The primary sequences of the END domain from type 1 EBV strains are highly homologous (>96% identity to B95-8). AG876, a type 2 strain, exhibits slightly lower sequence identity (79%), while the sequence identity of baboon and macaque LCV is significantly lower (41–50%). Interestingly, all hydrophobic amino acids which are an integral part of the dimeric interface are highly conserved between man and monkey viruses. Out of the eight residues, six are identical and two are highly similar (Fig 2B and 2C). This suggests that the dimer interface of the END domain is conserved in the EBNA-2 proteins of EBV and baboon and macaque LCV, and thus may play an important functional role. In addition, the program PSIPRED predicts 4 β-strands in similar positions for EBV and LCV END domains proposing that the dimer fold might be a conserved motif across species (Fig A in S1 Text).

**Fig 3:**
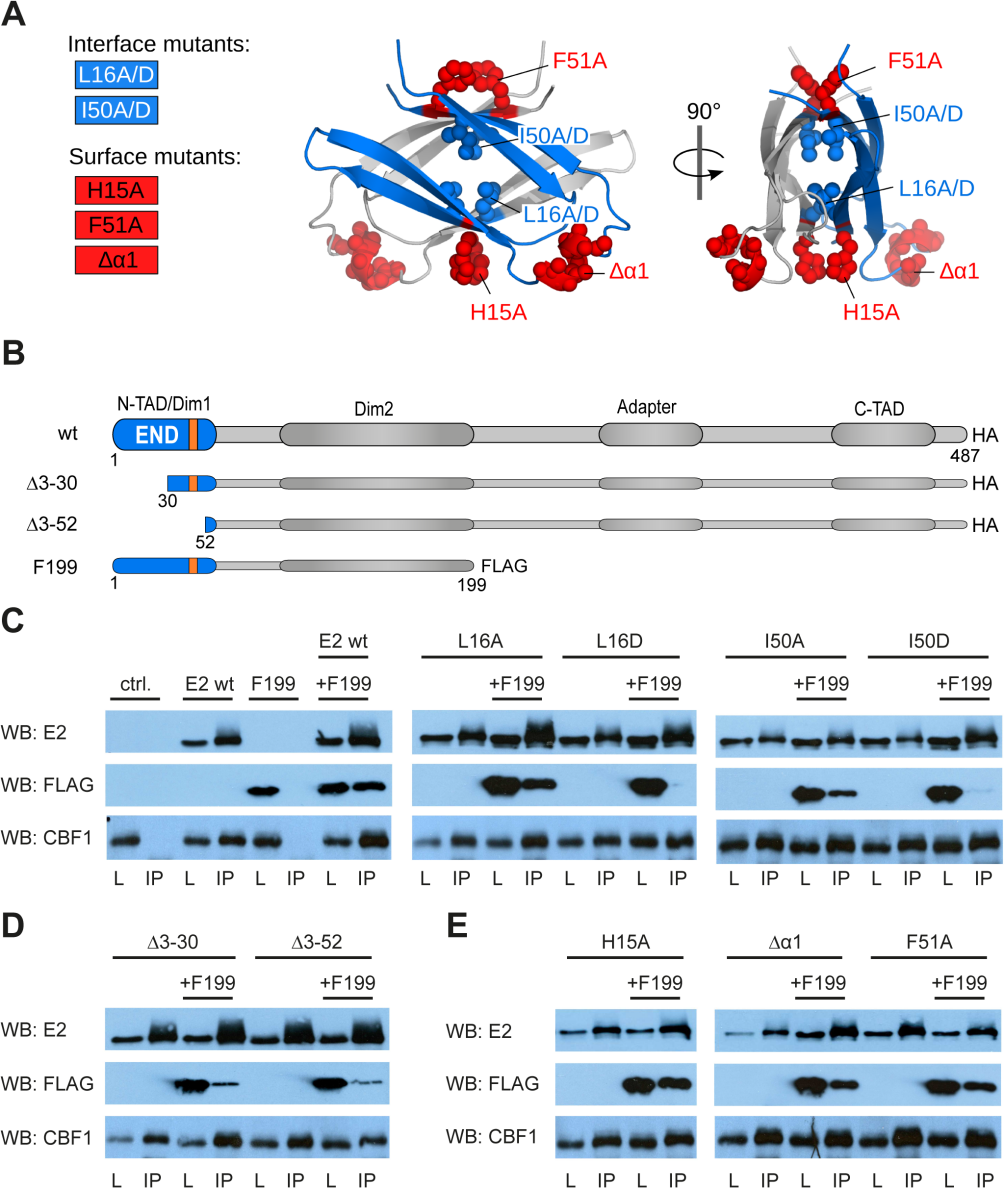
Amino acid substitutions of interface or surface residues within the END domain affect dimerization differentially. (A) Mutated interface (blue) and surface (red) residues highlighted as spheres on the structure of the END domain. (B) Schematic illustration of EBNA-2 and EBNA-2 mutants used in subsequent experiments. (The orange box represents the position of the α-helix). (C-E) HA-tagged EBNA-2 (E2 wt) or HA-tagged END domain mutants were co-expressed with FLAG-tagged EBNA-2 fragments truncated at aa199 (F199) in EBV negative DG75 B cells. Protein complexes were immunoprecipitated using HA-specific antibodies. The precipitates were detected in western blots either by EBNA-2 specific antibodies (E2) recognizing the EBNA-2 C-terminus (upper panel) or FLAG-specific antibodies recognizing F199 (middle panel) or CBF1/CSL specific antibodies recognizing endogenous protein (lower panel). Total lysates (L) correspond to 15% of the sample used for immunoprecipitation (IP). The following EBNA-2 mutants were used: (C) alanine or aspartic acid substitution mutants of residues Leu16 and Ile50 (L16A, L16D and I50A, I50D) residing in the hydrophobic interface of the END domain; (D) N-terminal deletion mutants Δ3–30 and Δ3–52; (E) alanine substitution of residues His15 or Phe51 (H15A and F51A) or deletion of the α-helix at position 35–39 (Δα1) on the surface of the END domain.

To determine the contribution of particular residues for END domain dimer formation, we designed mutations to disrupt specific interactions in the dimerization interface (interface mutants, Fig 3A). We replaced Leu16 and Ile50 by either alanine or aspartate as both residues are positioned directly at the interface and interact with the same residue in the other monomer. Replacement by aspartate was considered to introduce charge repulsion in the dimer interface and thus expected to strongly impair dimerization. Leu16 and Ile50 mediate important hydrophobic interactions and are completely conserved in all human and monkey sequences (Fig 2C). In a second set of mutations, we altered solvent-exposed residues at the surface of the END structure (surface mutants, Fig 3A), such as His15 and Phe51. We also studied an END domain variant where helix α1, residues 35–39, had been deleted (Δα1). These surface residues and helix α1 are not expected to be essential for dimerization but could mediate molecular interactions that might be required for functional activity.

The dimerization properties and structural integrity of the mutant END domains were characterized by SEC/SLS and NMR spectroscopy (Table 1 and Figs C and D in S1 Text). The interface mutants were more difficult to purify than the wild-type protein and are prone to aggregation as judged by SLS analysis. Due to the low solubility of mutant END domains, SLS was also performed on Z-tag fusion proteins to enhance solubility of the fusion proteins. The L16A mutant exists in equilibrium between an unfolded monomeric and folded dimeric state. The L16D, I50A, and I50D mutants are greatly destabilized leading to high molecular weight aggregates (SLS, Fig D in S1 Text) and could not be analyzed by NMR. The data suggest that interface mutations destabilize the dimerization interface and thus promote aggregation of monomeric END domains, as monomers would expose hydrophobic residues.

H15A yields homogeneous protein samples and is a dimer as indicated by SLS analysis (Fig D in S1 Text) and a well-dispersed NMR spectrum (Fig D in S1 Text). SLS data for F51A and Δα1 mutant END domains indicate the presence of dimer populations but also some aggregated species. This is further confirmed by NMR spectra, which are recorded at higher concentration and show the presence of dimeric and aggregated species in solution for these mutants (Fig D in S1 Text). Residue F51 is located at the surface of the END domain but contributes to the dimerization interface. Mutation to alanine may thus destabilize the dimer and lead to aggregation due to solvent exposure of the hydrophobic dimerization interface. Similarly, although removal of helix α1 does not globally disturb the fold and dimerization it may enhance aggregation at the concentrations used in NMR and SLS. NMR spectra clearly indicate the presence of folded dimer species for all surface mutants, i.e. H15A, F51A and Δα1. To further characterize these mutations, we analyzed their effect on dimerization of the full-length protein in cells (see below).

### Mutational analysis of END domain residues in full-length EBNA-2

As EBNA-2 has been reported to carry at least two domains implicated in dimerization (residues 1–58, i.e. the END domain, and residues 96–210), we tested whether mutants that abolish self-association of the END domain *in vitro* would also impair self-association of the full-length EBNA-2 protein [23, 24]. We expressed wild-type, deletion, surface and interface mutants as full-length EBNA-2 HA-tagged proteins and performed co-immunoprecipitation experiments in EBV negative DG75 cells [31] (Fig 3B–3E). For comparison we included HA-tagged mutants of EBNA-2 lacking amino acids 3–30 or 3–52 in our analyses (Δ3–30 and Δ3–52, respectively). All EBNA-2 mutants were expressed well and could be co-expressed with a FLAG-tagged EBNA-2 fragment encompassing amino acid 1–199 (F199). Co-immunoprecipitation studies using HA-specific antibodies indicated that all EBNA-2 mutants efficiently bound to endogenous CBF1. Both EBNA-2N-terminal deletion mutants were significantly impaired for self-association as has been reported previously (Fig 3D) [24]. The residual binding of Δ3–30 and Δ3–52 to F199 might be supported by the second self-association domain, comprising residues 96–210, which is still present in the F199 protein [23]. The self-association domain of a non-conserved region [27] is not present in F199 and thus cannot account for residual dimerization.

Next, we tested whether the interface mutants L16A, L16D, I50A and I50D can still mediate self-association with the EBNA-2 F199 fragment, which also harbors the END domain (Fig 3C, middle and right panel). While substitution of the Leu16 or Ile50 by alanine did not significantly affect F199 association, introduction of a negative charge by aspartic acid prevented self-association. These results confirmed the structural data indicating that hydrophobic residues facing each other across the dimer interface of the END domain are essential for EBNA-2 self-association. Surprisingly, Δ3–30 and Δ3–52 appeared to be less impaired than L16D and I50D.

In order to further validate the structural integrity of the END domain in the context of the complete EBNA-2 protein we tested the surface mutants H15A, Δα1 and F51A for association with F199 (Fig 3E). Consistent with the structural and biophysical data all surface mutants retained the capacity to self-associate, confirming that these residues are not essential for the dimerization of EBNA-2.

Nuclear localization and formation of nuclear speckles is a typical feature of EBNA-2 [32]. In order to analyze whether the END domain mutants had retained these features all EBNA-2 mutants were expressed in HeLa cells and the subcellular distribution of the EBNA-2 proteins was analyzed by confocal microscopy (Fig E in S1 Text). All mutants still showed strict nuclear localization, which typically excludes the nucleoli. Moreover, all mutants formed granular speckles, which are characteristic of wild-type EBNA-2 protein.

### The surface mutations H15A and Δα1 affect the function of the EBNA-2 protein

**Fig 4:**
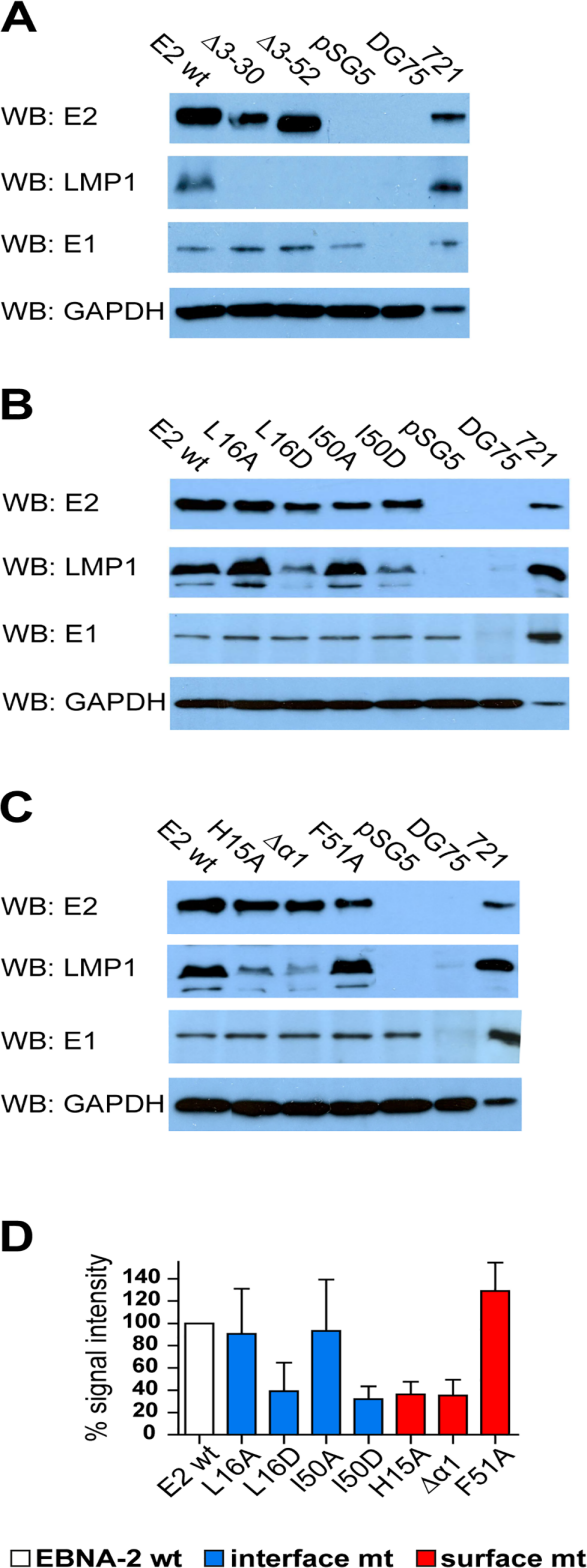
LMP1 activation by EBNA-2 requires dimerization, the surface residue His15, and the protruding α1-helix. 1x10^7^ EBV positive but EBNA-2 negative Eli-BL cells were transfected with 5 μg expression constructs for EBNA-2 wt, N-terminal deletion mutants (A), END interface (B) or END surface (C) mutants or the corresponding vector controls (pSG5). 30 μg of whole cell lysates of transfected cells were analyzed on western blots using EBNA-2, LMP1, EBNA-1 and GAPDH specific antibodies. Staining for EBNA-1 and GAPDH was used as loading controls. EBV negative (DG75: 30 μg of total cell lysate) and EBV infected LMP1 positive B cells (721: 5 μg total cell lysate) were used as controls. (D) The chemilumiscence signals were quantified by digital imaging using the Fusion Fx7 and the data are shown as % signal intensity relative to EBNA2 wt (100%). The bars represent the mean values of 4 independent experiments. Standard deviations are shown as error bars.

Based on previous work, EBNA-2 mutants impaired for dimerization were also severely impaired for activation of the viral target gene LMP1 [24]. In order to analyze the capacity of the EBNA-2 surface and interface mutants to activate the viral LMP genes we expressed EBNA-2 mutants in the EBV positive Burkitt's lymphoma cell line Eli-BL [33]. This B cell line exhibits a specific viral gene expression program where neither EBNA-2, nor EBNA-LP nor LMP proteins are expressed. By transient transfection of EBNA-2 expression constructs, endogenous LMP1 protein expression can be induced. We used this cellular system to measure the biological activity of EBNA-2 amino acid substitution mutants compared to N-terminal deletion mutants. The N-terminal deletion mutants, Δ3–30 and Δ3–52, which are expected to disrupt the END domain fold or delete it, are severely impaired for LMP1 activation, while the biological activity of the interface mutants L16A and I50A, which still self-associate, is comparable to wild-type EBNA-2 (Fig 4A and 4B). However, the functionality of the interface mutants L16D and I50D, which do not retain dimerization, is strongly attenuated. Notably, activation of LMP1 by the surface mutants H15A, F51A, and Δα1 is differentially affected. While F51A is unaffected, the activity of H15A and Δα1mutants is severely reduced (Fig 4C). EBNA-2 and all EBNA-2 mutants were expressed well in Eli-BL. Thus, the distinct biological activity of the mutant EBNA-2 proteins is not due to differential expression levels (Fig 4D).

**Fig 5:**
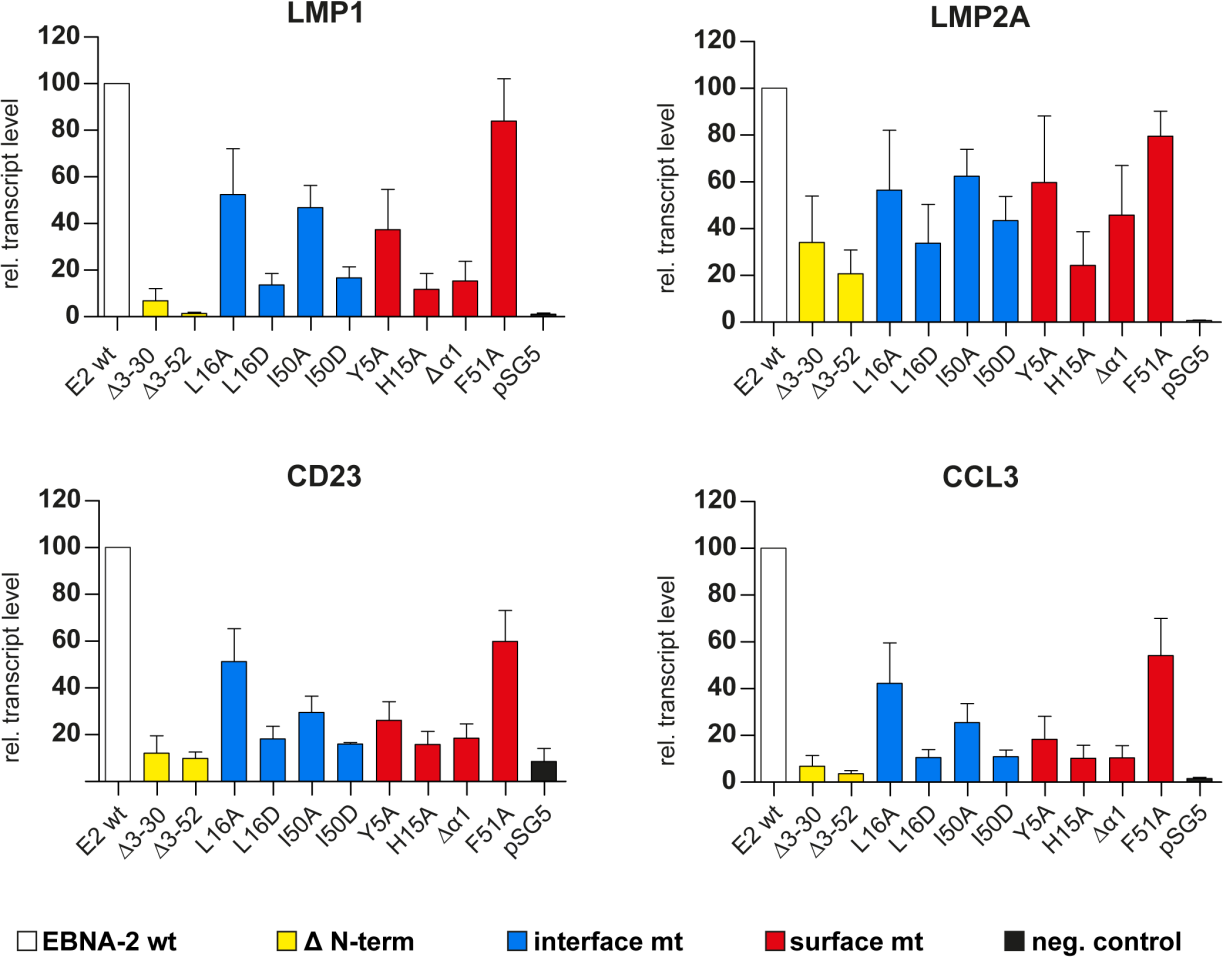
Transcriptional activation of endogenous viral and cellular target genes by END domain mutants. 1x10^7^ Eli-BL cells were transfected with expression constructs for EBNA-2 wt, N-terminal deletion mutants, END domain mutants or the corresponding control vectors (pSG5). Relative transcript levels of the viral LMP1 and LMP2A gene or the cellular CD23 or CCL3 genes were determined by real-time RT-PCR. Transcript levels were normalized to actin transcript levels. EBNA-2 activation was set to 100% and the data are shown as mean values of four independent experiments. Error bars indicate the standard deviations.

In order to analyze the capacity of all EBNA-2 mutants to induce endogenous transcripts we selected two viral, LMP1 and LMP2A, and two cellular target genes, CCL3 and CD23, for quantitative RT-PCR analyses in Eli-BL (Fig 5). These four genes all carry functional CBF1/CSL binding sites in their promoter region within less than 500 base pairs upstream of the transcription start site [34, 35]. The LMP1 promoter is controlled by a complex network of transcription factors that includes CBF1/CSL. However, although CBF1/CSL enhances transactivation by EBNA-2, the LMP1 promoter is unique since it can still be activated by EBNA-2 to up to 50% in the absence of CBF1/CSL [36] (and our unpublished data). In contrast, the LMP2A promoter carries two adjacent CBF1/CSL sites which are essential for EBNA-2 transactivation. Activation of the two cellular genes CCL3 and CD23 is strictly CBF1/CSL dependent [37–39]. Compared to wild-type EBNA-2, all END domain mutants, even those that still dimerize in cells, showed some loss of activity indicating that the integrity of this domain is critical for EBNA-2 function. The surface mutant F51A appears to be affected the least. Neither Δ3–30 nor Δ3–52 could efficiently activate any of the four genes. LMP1 induction was impaired the most, while activation of LMP2A is the least sensitive.

In parallel we studied the activity of the viral C promoter and the endogenous EBNA-2 transcript levels after transfection. C promoter transcript levels were close to detection limits and were not modulated by either EBNA-2 or EBNA-2 mutants. Endogenous EBNA-2 transcript levels were undetectable and could also not be induced. Thus, we can exclude that endogenous EBNA-2 in Eli-BL interferes with our assay in Eli-BL (Fig F in S1 Text). It appears that the END domain is critical not only for LMP1 transactivation but rather is required in a universal manner for transactivation of unrelated genes although to different extent.

**Fig 6:**
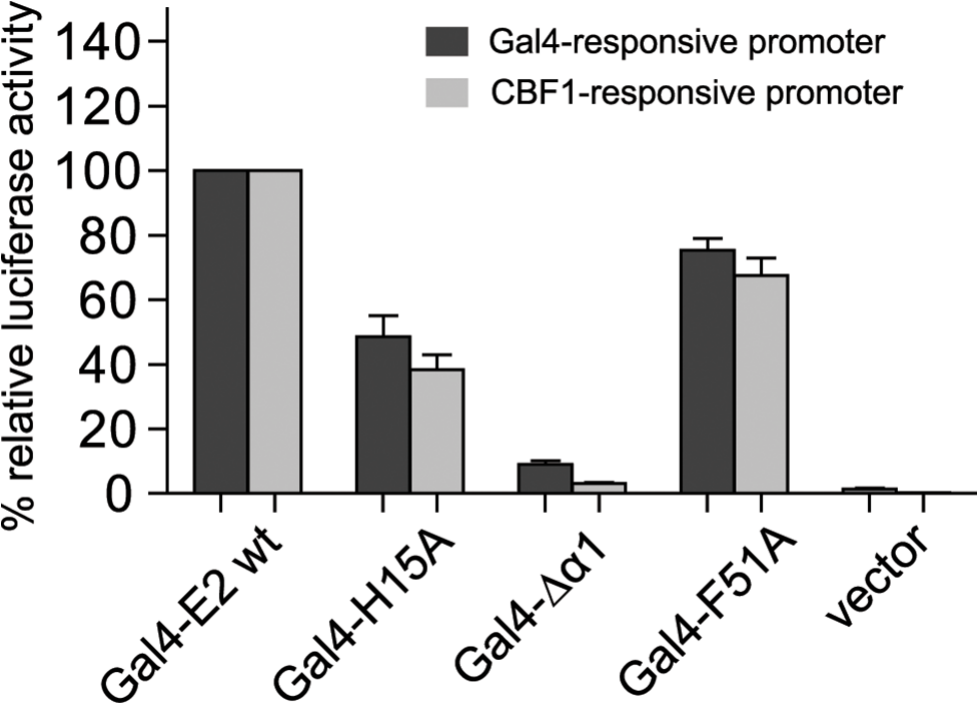
GAL4 DNA-binding fusion proteins of the END domain surface mutants H15A and ΔΔ1 have lost the capacity to activate GAL4-responsive and CBF1-responsive promoters. 5x10^6^ EBV negative DG75 cells were co-transfected with 5 μg of expression constructs for the GAL4 DNA-binding domain fused to EBNA-2 (GAL4-E2 wt) or EBNA-2 END domain mutants with either 5μg GAL4-responsive or CBF1-responsive promoter luciferase constructs plus 0.5 μg of Renilla luciferase construct. EBNA-2 activation of the reporter constructs was set to 100% and the data are shown as the mean of three independent experiments done in triplicates. Error bars indicate the standard deviation.

In order to prove that the END domain surface has a general impact on the transactivation capacity of the EBNA-2 protein we performed promoter reporter luciferase assays using Gal4 DNA-binding domain fusion proteins and two distinct promoter reporter constructs which either carried 10 GAL4 binding sites or 12 CBF1 binding sites to recruit GAL4 EBNA-2 (Fig 6). GAL4 EBNA-2 was efficiently recruited to both promoters and activated luciferase expression. The GAL4 EBNA-2 H15A mutant had lost more than 50% of its transactivation capacity on both luciferase constructs. The biological activity of GAL4 EBNA-2 Δα1 was almost completely abolished. Again the surface F51A mutant was affected the least.

In summary, EBNA-2 END domain mutations that do not affect dimerization are severely impaired for transactivation of endogenous target genes as well as artificial promoter reporter constructs. Loss of function was most pronounced for END domain deletion mutants and was almost as strongly observed with the surface mutants H15A and Δα1. The dramatic loss of function seen in mutants – that still dimerize, properly localize to the nucleus, and bind to CBF1 – suggests that the END domain not only promotes dimerization of EBNA-2 but conveys additional critical functions.

## Discussion

### The EBNA-2 END domain represents a novel dimerization motif

Here, we report the first three-dimensional structure information for the EBNA-2 protein. The N-terminal region of EBNA-2 represents a specific dimerization domain designated END (EBNA-2 N-terminal Dimerization) domain. The dimer is stabilized by anti-parallel interactions of β4-β4’ and β2-β2’, which generate a strong hydrophobic interface which stabilizes the dimer. In fact, dimerization via hydrophobic interfaces of diverse structures is a frequent feature of small dimers (<100 aa per monomer) [40]. However, to our knowledge the specific fold of the END domain dimer is novel. Notably, the hydrophobic residues which form the dimerization interface are completely conserved in EBV and rhesus LCV sequences. We thus expect that the dimerization by the END domain is conserved in all EBV sequences and most likely also in macaque and baboon EBNA-2 orthologs.

To probe the dimerization interface we generated END domain mutants which affect residues in the dimer interface. Mutation of these interface residues were indeed found to disrupt the fold of the END domain and/or lead to aggregation of recombinant protein. For further analysis, all END domain mutants were expressed as full length EBNA-2 protein in human B cells and tested for self-association and transactivation of endogenous target genes. While self-association of the EBNA-2 L16A and I50A interface mutants was marginally impaired, self-association of L16D and I50D was close to or below detection levels. Surprisingly, even the N-terminal deletion mutants (Δ3–30 and Δ3–52) exhibited residual binding activity stronger than L16D and I50D. Potentially the second dimerization domain (Dim2, Fig 1A) could be unmasked in the absence of the END domain. Or, single amino acid substitutions in the hydrophobic core may cause non-physiological aggregation-states of EBNA-2 and impair protein function even stronger than loss of the END domain.

Our data provide convincing evidence that the END domain is a conserved dimerization motif for the full-length EBNA-2 protein. As the END domain is separated from the rest of the EBNA-2 protein by an extended poly-proline hinge region, we suggest that the END domain acts as an independent module that mediates self-association of the entire protein.

EBNA-2 is recruited to DNA by adapters like CBF1/CSL but might require at least two factors to which it binds simultaneously to activate viral target genes. So, the viral LMP2A promoter carries two functional CBF1/CSL binding sites, while the LMP1 promoter requires PU.1 and CBF1/CSL for efficient activation by EBNA-2 [36, 41, 42]. By using CBF1/CSL as a DNA adapter, EBNA-2 mimics the activated Notch receptor which also is recruited to DNA by CBF1/CSL. Interestingly, Notch dimers frequently use paired CBF1/CSL1 binding sites in the cellular genome [43] which might also be used by EBNA-2 dimers. In the cellular genome, EBNA-2 binds preferentially to enhancers which can be located remote from the promoter of the regulated genes [44]. Thus, it may be proposed that dimerization promotes higher order protein complex assembly that bridges promoter and enhancer regions.

### The END domain might provide an interaction surface that is critical for transactivation

According to the NMR and SEC/SLS analyses all surface mutants of the END domain are folded and comprise dimeric species, although F51A and Δα1 have a tendency to aggregate. In B cells, the full-length surface mutants EBNA-2 H15A, Δα1, and F51A mutants self-associate, further corroborating the in vitro data. Notably, transactivation of target genes by the surface mutants H15A and Δα1 was severely reduced to similar levels observed for aspartic acid interface mutants, which abolish self-association. This indicates that the effects onto the functional activity are not due to impaired dimerization but suggest that these residues may be involved in additional intra- or intermolecular molecular interactions.

We directly compared the different END domain EBNA-2 mutants for their capacity to induce either LMP1 protein expression or endogenous LMP1, LMP2A, CCL3, or CD23 transcript levels in Eli-BL cells. These four genes share functional CBF1 binding motifs but rely on these motifs to varying degrees. Importantly, all END domain mutants retain the capacity to bind to CBF1. We find that the residual self-association of the two N-terminal deletion mutants (Fig 3D) is not sufficient to restore the biological activity of the mutants to wild-type levels (Figs 4 and 5). Although LMP1 and CCL3 induction are affected the most, all mutants produce similar patterns of loss of activity for all genes we have tested. Since we did not observe a gene specific phenotype for any of the mutants, a single so far unknown factor could interact with the END domain of EBNA-2 and be required for the activation of each of the four target genes. In EBV infected B cells, the EBNA-LP co-activator of EBNA-2 could be a candidate factor to play this role. However, since EBNA-LP is not expressed in EBV negative DG75 cells and neither expressed nor induced by EBNA-2 in Eli-BL cells [45], EBNA-LP can be excluded in our setting. At this point of our studies we speculate that basic mechanisms of transcriptional activation by EBNA-2 are impaired in the surface mutants H15A and Δα1.

In the past, multiple transactivation domains (TADs) have been defined by generating chimeras of protein fragments of interest and an unrelated DNA binding domain. These chimeras were tested for their activity to induce artificial promoters recruited by the DNA binding domain [46]. Most of the TADs, which scored positive in these assays, were enriched for hydrophobic or acidic amino acids or a 9aa TAD sequence motif [47]. In retrospect it was found that TADs not only bind to general factors of the transcription machinery, but also confer contact to components of the mediator, the SAGA complex or the chromatin remodeling machinery. Most TADs appear to be intrinsically unstructured. However, in complex with their cognate binding partners they may fold into specific structures which mediate protein-protein interactions (reviewed in [48]). In contrast to the acidic C-TAD of EBNA-2, which is intrinsically unstructured and attains a stable secondary structure only upon complex formation with cellular proteins [21], the END domain appears to be a non-typical TAD. In the absence of any cognate cellular binding partner the END domain folds into a well-defined rigid dimeric globular structure.

Taken together our structural and mutational analysis suggests that the dimerization by the END domain provides a surface that is critical for transactivation of target genes, for example, by exposing His15 and the α1-helix. Since all loss-of-function mutants interfere with activation of all genes that were tested, the END domain is likely to interact with candidate proteins which could be critical for transactivation at multiple steps.

EBNA-2 expression is a hallmark of B cell lymphomas arising in immunocompromised patients and considered to drive the proliferation of these cells. The END domain has a strong impact on the biological activity of EBNA-2 and thus it should be considered as a potential drug target for small molecules [24, 26]. The END domain forms a novel, highly stable parallel dimeric fold, which is stabilized by conserved hydrophobic interactions. Importantly, our *in silico* searches for cellular protein sequences or related folds similar to the END domain did not reveal any homologous cellular domains suggesting that the END domain is a unique structure that evolved in lymphocryptoviruses and thus is virus specific. Our future studies will focus on the identification of potential proteins which bind to the END domain and require His15 or the α-helix for protein interactions. The dimerization or the suggested binding surface of the END domain might be targeted by small molecules to impair EBNA-2 activity for potential therapeutic intervention.

## Material and Methods

### Plasmids

The design of constructs for structural and biochemical studies was guided by secondary structure prediction (PSIPRED) [28]. Residues 1–58 of EBNA-2 (Strain B95-8; Uniprot: P12978) were cloned into a modified pET-24d expression plasmid following standard restriction digest procedures. The vector contained a Z-tag, as well as a 6xHis-tag to facilitate purification. The Z-tag is a 125 amino acid protein tag based on protein A from *Staphylococcus aureus* and is known to enhance the solubility of fusion proteins [49]. Both of these N-terminal tags could be removed by proteolytic cleavage using tobacco etch virus (TEV) protease. For cloning purposes and efficient TEV protease cleavage the final protein construct contained four additional residues at the N-terminus (Gly-Ala-Met-Glu). Mutations to study the functional importance of the END domain were introduced by overlap extension (also known as two-step) PCR. In brief, mutation primers were used in combination with the original forward or reverse primers in a first round of separate PCR experiments. The purified products were then combined and used as the template for a second round of PCR using only the original forward and reverse primers. Restriction digestion and ligation of the final product yielded expression plasmids in a similar way to the original construct. Mutant END domains were expressed and purified in similar fashion as the wild-type protein. For expression studies in mammalian cells all END domain mutant gene fragments were sub-cloned into pAG155, to generate EBNA-2 carrying an HA tag at the C-terminus of full-length proteins by conventional cloning techniques [24]. In order to express GAL4 EBNA-2 fusion proteins the GAL4 DNA binding domain (DBD) gene fragment was added to the 5’ end of the EBNA-2-HA ORF. Luciferase promoter reporter gene assays were performed using the Promega dual luciferase assay system. The CBF1 reporter (pGa981-6) carries 12 CBF1 binding sites [50] and the GAL4 (Gal4 tk-Luc) responsive reporter construct carries 10 GAL4 binding sites. For normalization the pRL-PGK Renilla Luciferase construct was used. The integrity of all expression plasmids was confirmed by sequencing.

### Protein expression and purification of the EBNA-2 END domain

Recombinant proteins were expressed in *Escherichia coli* BL21 (DE3). Using kanamycin for selection, one colony was picked from a fresh transformation plate to inoculate a 5 mL pre-culture in lysogeny brothmedium. The pre-culture was used to start larger culture volumes of unlabeled LB, or minimal M9 media for expression of isotope-labeled proteins. For production of ^13^C and ^15^N-labeled protein samples [U-^13^C]-D-glucose and ^15^NH_4_Cl were included as the sole carbon and nitrogen sources, respectively. Cultures were grown at 37°C until the optical density reached 0.8 and then, after cooling to 20°C, induced overnight (16 h) by addition of 0.5mM isopropyl β-D-1-thiogalactopyranoside. Cells were harvested by centrifugation (8000 g, 20min) and disrupted by pulsed sonication (6 min, 30% power, large probe, Fisher Scientific model 550) in lysis buffer (20 mM TRIS pH 7.5, 300 mM NaCl, 10 mM imidazole, and 0.02% NaN_3_), containing protease inhibitors, DNase, lysozyme, and 0.2% IGEPAL. After centrifugation and filtering the lysate was passed three times over Ni-NTA agarose resin (Qiagen) in gravity-flow columns (Bio-Rad). Bound protein was washed extensively with the lysis buffer, the lysis buffer containing no IGEPAL, and lysis buffer with high salt NaCl (1 M) or imidazole (30 mM) concentrations. The protein was eluted with the elution buffer (20 mM TRIS pH 7.5, 300 mM NaCl, 300 mM imidazole, and 0.02% NaN_3_). The eluted protein was buffer exchanged into TEV cleavage buffer (10 mM NaP pH 7.5, 150 mM NaCl, 1 mM DTT, and 0.02% NaN_3_). TEV protease was added to a molar ratio of 1:10, protease to recombinant protein, and incubated overnight at 4°C. To efficiently remove TEV protease and the cleaved off solubility tag, the sample was passed over an ion-exchange column (Resource Q, GE Healthcare) which was equilibrated with the buffer (20 mM sodium phosphate, pH 6.9, 20 mM NaCl, and 0.02% NaN_3_). The protein was eluted from Resource Q column with a NaCl gradient (0–0.5M over 60 ml). Additionally, a last purification step was implemented and included size-exclusion chromatography (HiLoad16/60, Superdex 75, GE Healthcare). The size-exclusion column was equilibrated and run in a buffer appropriate to subsequent studies.

### Nuclear magnetic resonance (NMR) and structure determination

NMR experiments were performed on Bruker instruments operating at a field-strength corresponding to a proton resonance frequency of 500, 600, 750, 800, and 900 MHz equipped with pulsed field gradients and cryogenic probes (except at 750 MHz). Spectra were generally recorded at 323K (50°C) on protein samples (1 mM) in20 mM sodium phosphate, pH 6.9, 20 mM NaCl, and 0.02% NaN_3_. Spectra were processed with NMRPipe [51] and analyzed in NMRView [52] and Sparky 3.

For assignment of backbone amides and side-chain signals the following multidimensional heteronuclear experiments were acquired [53]: ^1^H,^15^N-HSQC, ^1^H,^13^C-HSQC, HNCA, HNCACB, CBCA(CO)NH, (H)CC(CO)NH-TOCSY, H(C)CH-TOCSY, and HCC(H)-TOCSY. Assignment of aromatic protons was accomplished by two-dimensional (HB)CB(CG,CD)HD and (HB)CB(CG,CD,CE)HE spectra. Stereospecific assignment of the methyl groups in leucine and valine residues was achieved by partial ^13^C-labeling and by observing the presence or absence of a hydrogen-carbon J-coupling in a 2D ^1^H-^13^C HSQC [54]. Distance restraints were derived from three-dimensional NOESY experiments: ^1^H,^15^N-HSQC-NOESY,^1^H,^13^C-HMQC-NOESY (for both the aliphatic and the aromatic region), and ^13^C-edited-^15^N/^13^C-filtered NOESY (aliphatic region). Denaturation and refolding of the END dimer was required for measurement of the intermolecular NOEs. This was accomplished by taking equimolar amounts of unlabeled and double labeled (^15^N, ^13^C) protein and adding 8M urea. The mixture was heated to 80°C for 10 min and then dialyzed twice against NMR buffer at 4°C. Importantly, appropriate samples were lyophilized and dissolved in pure D_2_O to increase sensitivity of several experiments, and to simplify spectral analysis.

Automated NOESY assignment and derivation of distance restraints was performed using CYANA v3.0 [55]. Dihedral restraints were obtained with TALOS+ [56], using assigned chemical shifts as input, and inspected manually to remove less reliable predictions. The final structure calculations in ARIA v2.2 [57] included refinement in explicit water and activation of a non-crystallographic two-fold symmetry constraint. Out of one hundred calculated structures, ten models were selected as a representative ensemble based on low energy and restraint violations. Analysis of structure quality and restraint violations was performed with iCing including PROCHECK [58] and WHATCHECK [59]. Figures and structure ensemble alignment were prepared in Pymol v1.5 [60].

^1^H,-^15^N heteronuclear NOEs were measured at 318K on a 500 μM ^15^N-labeled sample (750 MHz proton Larmor frequency) as described previously [61], and analyzed in NMRView. The secondary chemical shift analysis was also done in NMRView. Hydrogen-deuterium exchange experiments were performed by NMR to detect solvent protected backbone amide protons. A ^1^H,^15^N-HSQC was recorded on a lyophilized protein sample 10 min after dissolving it in D_2_O, and compared to a reference spectra in H_2_O. Both spectra were recorded at 313K to reduce the amide proton exchange rates with the solvent. Any residual signals observed above noise were considered indicative of solvent protected amide protons.

### Structural similarity search

A BLAST sequence search of the Protein Data Bank (PDB) generated no hits with reasonable E-values (< 1) or domains with structural similarities to the END domain. The fold of the END domain was further compared to previously determined protein structures deposited in the PDB using the DALI server as well as PDBeFold, available from EMBL/EBI. Interestingly, the DALI server only returned low-scoring hits for the complete dimer with relatively high RMSD values and low sequence identity. The structural superpositions of the END domain with the top twenty hits were manually examined, without the discovery of any similar folds. The most commonly matched structural feature of the END domain was the large anti-parallel beta-sheets (β1-β4-β4’-β1’), while the rest of the dimer and the ordering of the beta-strands, never exhibited an adequate fit. Likewise, PDBeFold produced no hits with reliable scores for the END monomer. Top hits only matched two out of the five secondary structure elements, and visual inspection confirmed lack of conserved structures. In conclusion this lack of similar structures strongly suggests that the END domain is of a novel fold and that this is the first structural determination of this viral dimerization motif.

### Static light scattering (SLS)

SLS was measured with a Malvern-Viscotekinstrument (TDA 305) connected downstream to an Äkta Purifier equipped with an analytical size-exclusion column (Superdex 75 10/300 GL, GE Healthcare). Samples were run at a concentration between 150 and 400 μM in a running buffer containing 20 mM NaP pH 6.9, 20 mM NaCl, and 0.02% NaN_3_. Elution profiles were collected for 30 min with a flow rate of 1 mL/min. Data were collected using absorbance UV detection at 280 nm, right angle light scattering (RALS) and refractive index (RI). The molar masses of separated elution peaks were calculated using OmniSEC software (Malvern). As standard for calibration, 4 mg/mL Bovine Serum Albumin (BSA) was used prior to all experiments and the change in refractive index with respect to concentration (dn/dc) was set to 0.186 mL/g [62].

### Cell culture, transfection conditions, and luciferase reporter assays

DG75 [31], Eli-BL [33], and 721 [63] cells were maintained in RPMI 1640 medium supplemented with 10% fetal calf serum, 100 U/mL penicillin, 100 μg/mL streptomycin and 4 mM glutamine at 37°C in a 6% CO_2_atmosphere. For transfection, 5x10^6^DG75 or 2x10^7^ Eli-BL cells were electroporated in 250 μL Optimem medium at 240 V and 975 μF using the Genepulser II (Bio-Rad) and allowed to recover in 10 mL of cell culture medium for 24 h. Luciferase promoter reporter gene assays were performed using the dual luciferase assay system (Promega) according to the manufacturer's instructions. Results obtained for firefly luciferase activity were normalized to Renilla luciferase activity.

### Immunofluorescence microscopy

HeLa [64] cells were cultivated in DMEM supplemented with 10% fetal calf serum, 100 U/mL penicillin, 100 μg/mL streptomycin and 4 mM glutamine at 37°C in a 6% CO2 atmosphere. Cells were transfected with a mixture 1.5 μg of EBNA2 expression plasmids and 4 μg polyethylenimine (Sigma) in the presence of Optimem (Gibco). After 4 h, the medium was replaced with cell culture medium and cells were allowed to recover for 24 h and subsequently cultured for 24 h on cover slips. The cells were fixed with 2% paraformaldehyde (PFA) at RT for 15 min and subsequently permeabilized with PBS/0.15% TritonX-100 3 for5 min at RT. All samples were blocked with 1% BSA/0.15% glycine 3x for 10 min and incubated with the EBNA-2 specific antibody (R3) over night at 4°C. Cells were washed with PBS for 5 min, with PBS/0.15% TritonX-10 for 5 min, with PBS 5 min, blocked with PBS/1% BSA/0.15% glycine for 7 min and incubated with Cy3-conjugated goat anti-rat immunoglobulin (Jackson Immuno Research) in the dark for 45 min at RT. Cells were washed again with PBS/0.15% TritonX-100, and with PBS and stained with 0.1μg/ml 4',6-diamidino-2-phenylindole (DAPI) (Sigma) for 90sec and washed with PBS. Samples were embedded in fluorescent mounting medium (DakoCytomation). Confocal microscopy was performed on a Leica LSCM SP5 microscope equipped with 405 nm, 488 nm, 561 nm and 633 nm lasers. Images were taken with an objective HCX PL APO 63/1.4 objective and an electronic zoom of 3.6. Laser line 405 nm (DAPI) and 561 nm (Cy3) were used for image acquisition. Detection settings were carefully chosen to exclude spill-over of DAPI and Cy3 fluorescence.

### Immunoprecipitation, immunoblot assays and antibodies

For immunoprecipitation studies DG75 cells were lysed in 1% NP-40 buffer (10 mM TRIS pH7.4, 1 mM EDTA, 150 mM NaCl, 3% Glycerol, 1x complete protease inhibitor tablets (Roche)). The lysates were submitted to immunoprecipitation and total cell lysates and immunoprecipitates were analyzed by immunoblotting. For direct immunoblotting of Eli-BL cells they were lysed in RIPA buffer (50mM TRIS pH7.5, 150mM NaCl, 1% Igepal, 0.1% SDS, 0.5% Na-deoxycholate, 1x complete protease inhibitor tablets (Roche)) for 1 h and sonicated for 10 min (30s on, 30s off) at 230 V using a Bioruptor (Diagenode). Immunoblot assays were performed as described previously [38]. HA (3F10, Roche) and Flag (M2, Sigma) specific antibodies were obtained from commercial sources. The EBNA-2 (R3) [65], the EBNA-1(1H4) [66] and the LMP1 specific monoclonal antibodies (S12) [67] are published. Chemilumiscence signals of immunoblots were quantified by digital imaging using the Fusion Fx7.

### Real time RT-PCR assays

Total RNA was extracted from 1x10^7^ transfected Eli-BL cells 24 h post-transfection using the Qiagen RNeasy Mini Kit and cDNA was synthesized from 2 μg of RNA using the High-Capacity cDNA Reverse Transcription kit (Applied Biosystems) according the manufacturer´s protocol. qPCR of the transcripts was performed on a LightCycler 480 SYBR Green I Master (Roche) and the data were processed with the LightCycler 480 software (version 1.5.0.39, Roche). A total of 1/80 of cDNA product was used for amplification of actin and 1/40 of cDNA for all other genes. Cycling conditions were 10 min at 95°C and 45 cycles of 3 s at 95°C, 10 s at 60 or 63°C, and 20 s at 72°C on a 96-well thermal block. PCR products were validated by melting curve analysis and agarose gel electrophoresis. Quantification was based on standard samples of known concentration and standard curves for each primer pair. Primer pairs for RT-PCR were selected by Primer3 software All pairs were chosen to support amplification across intron borders. Primers were GGTGTTCATCACTGTGTCGTTGTC and GCTACTGTTTTGGCTGTACATCGT for LMP1 [68], ATGACTCATCTCAACACATA and CATGTTAGGCAAATTGCAAA for LMP2A [69], CTGGGACACCACACAGAGTC and GACACCTGCAACTCCATCCT for CD23, ATGCAGGTCTCCACTGCTG and TTTCTGGACCCACTCCTCAC for CCL3, AGATCAGATGGCATAGAGAC and GACCGGTGCCTTCTTAGGAG for C promoter usage, GCTGCTACGCATTAGAGACC and TCCTGGTAGGGATTCGAGGG for EBNA-2 [70], and GGCATCCTCACCCTGAAGTA and GGGGTGTTGAAGGTCTCAAA for actin.

### Accession numbers

Atomic coordinates of the END domain have been deposited at the Protein Data Bank (PDB) with accession code 2N2J. Experimental NMR distance restraints have been deposited at the Biological Magnetic Resonance Bank (BMRB) with accession number 19390.

## Supporting Information

S1 Text Includes Figs A-F. (PDF)
