# Supplementary material for: The EBNA-2 N-Terminal Transactivation Domain Folds into a Dimeric Structure Required for Target Gene Activation
Source: PLoS Pathog. 2015 May 29;11(5):e1004910. doi: 10.1371/journal.ppat.1004910 (PMC4449002; doi:10.1371/journal.ppat.1004910)
Supplement: S1 Text — (PDF) [file ppat.1004910.s001.pdf]

Supporting Material

S1 Text

Methods

Biophysical characterization of EBNA-2 END domain mutants. Wild-type and H15A END domains without a Z-tag elute as a single molecular species with the experimental molecular weights (MW) close to that of a theoretical dimer (13.3 kDa, Fig. C, left). L16A elutes with the same volume as a monomer but exhibits an apparent MW between a monomer and a dimer. This is in line with the observation by NMR that this mutant protein is in equilibrium between a monomeric and dimeric state (see Fig. D). Note, that the retention time of H15A in size-exclusion chromatography (marked with an asterisk in Fig. C) is similar to the retention time of a monomer, while the molecular weight, calculated from RALS and RI data, corresponds to a dimer. This apparent discrepancy likely results from a temperature difference between the column (stored at 4 °C) and the RALS detector (kept at 30 °C). At 4 °C (retention on the column) the monomer-dimer equilibrium is shifted towards the monomer, whereas at 30 °C, it is shifted towards the dimer. F51A and $\Delta\alpha 1$ elute as dimers mixed with aggregated states. L16D, I50A, I50D analyzed without a Z-tag displayed aggregation behavior, i.e. eluted early, and yielded no reliable MW values.

In the presence of a Z-tag, wild-type and H15A END domains exist as dimers. In this context, the apparent molecular weight of the L16A mutant was closer to a theoretical monomer (23.4 kDa, Fig. C, right). Its elution volume was also slightly higher, as expected for a monomer. Chromatograms of L16D, I50A, and I50D indicate the presence of aggregation. This likely result from the destabilization of the dimer interface that lead to monomers with exposed hydrophobic surface, rendering these mutants prone to aggregation. This notion is further supported by the lack of self-association for these protein constructs as observed in the co-immunoprecipitation experiments described in the main text. Due to the low resolution no reliable MW values could be extracted from these plots. In the presence of a solubilizing Z-tag, F51A and $\Delta\alpha 1$ still exhibit aggregation with the presence of folded dimer states. This is consistent with the results of our co-immunoprecipitation experiments as well as with the NMR data (presented in Fig. D). As expected, F51A and $\Delta\alpha 1$ do self-associate *in vivo* and *in vitro*.

Figure A

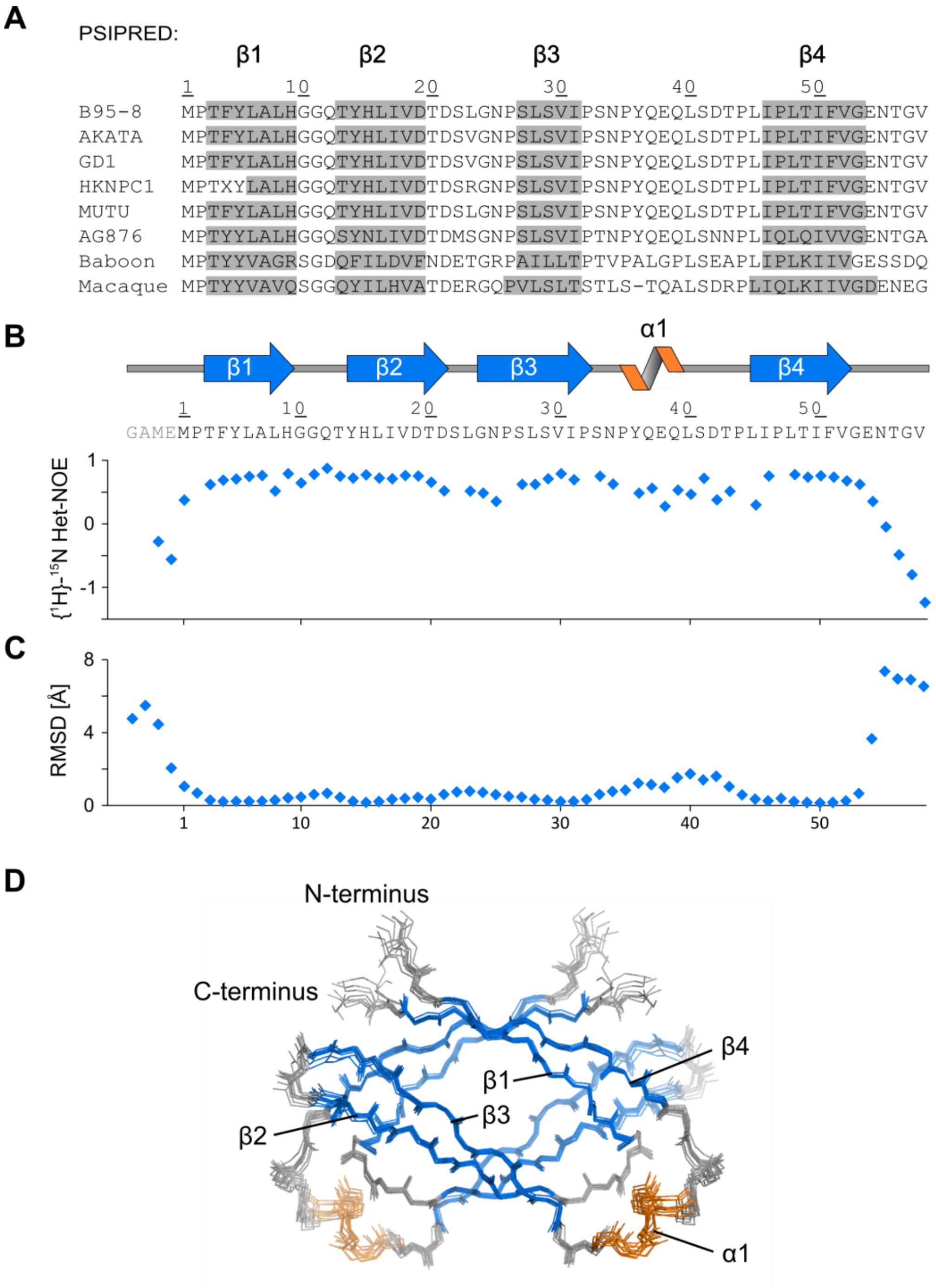

Figure A. Dynamics of the END domain and secondary structure prediction. (A) Secondary structure prediction of END domain orthologs in LCV and EBV. (B) $\{^1H\}$-^{15}N heteronuclear NOE data indicate that the C-terminus of the END domain is flexible. (C) The RMSD of the backbone atoms (N, Cα and CO) in the calculated ensemble of ten low energy structures. Please note the correlation in increasing RMSD to lower heteronuclear NOE values. (D) Ensemble of ten low-energy NMR models, displayed as a wire model of the protein backbone.

Supporting Material

Figure B

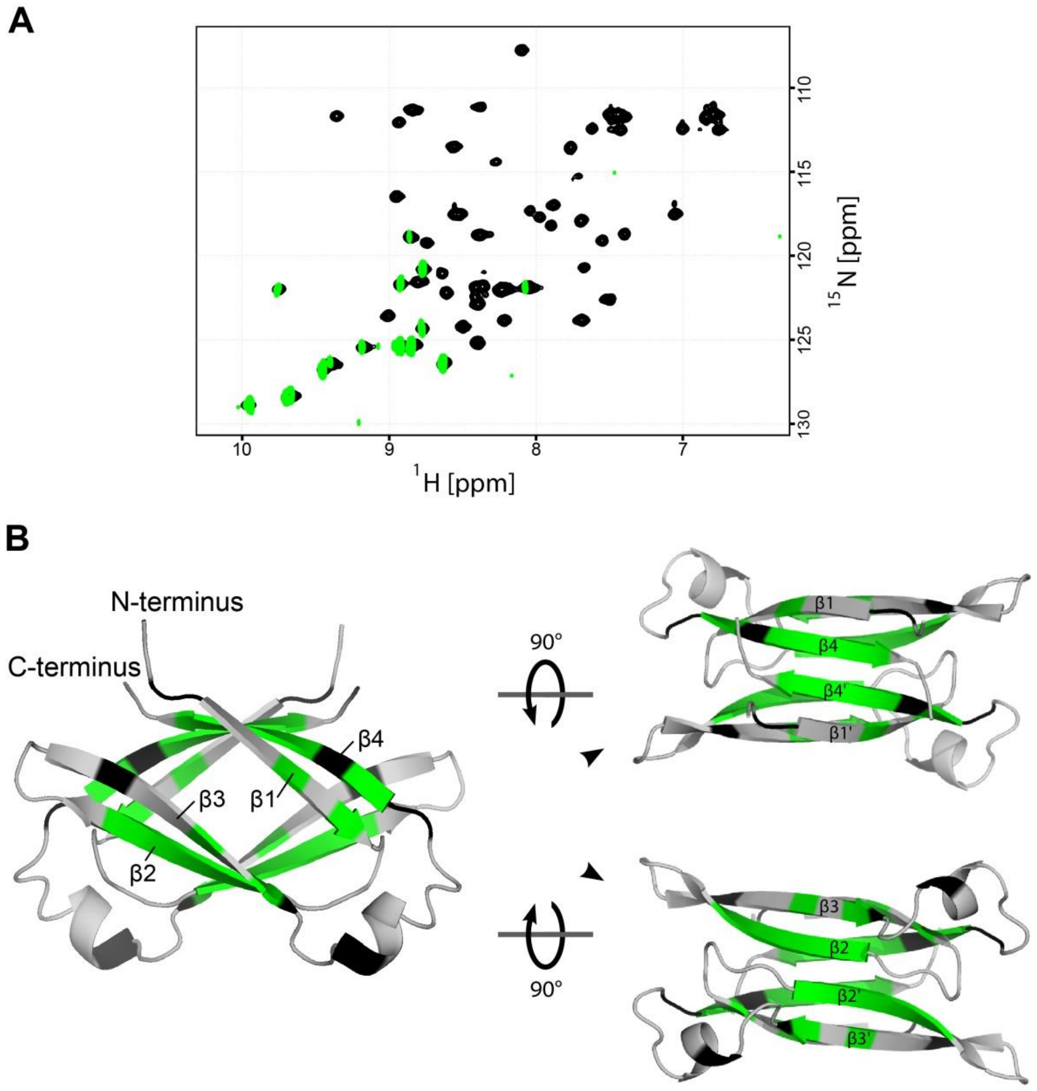

Figure B. Hydrogen-deuterium exchange experiments by NMR. (A) $^{1}H,^{15}N$-HSQC spectrum of wild-type END in H_2O (black) superimposed on a spectrum acquired 10 min after a lyophilized sample was dissolved in D_2O (green). Retained signals correspond to the following residues: Leu6, Leu8, Tyr14, His15, Leu16, Ile17, Val18, Asp19, Ser29, Ile31, Leu45, Ile46, Leu48, Thr49, Ile50, and Phe51. (B) The result indicates that these backbone amide protons are solvent protected (green) and, presumably, participating in hydrogen bonds of secondary structure elements. Each inter-monomer contact (β4-β4' and β2-β2', right) is remarkably stable, while amides of loops and α1 are solvent exposed and/or flexible (gray). Importantly, these results corroborate the calculated NMR structure. Proline residues (no amide proton) are colored black.

Supporting Material

Figure C

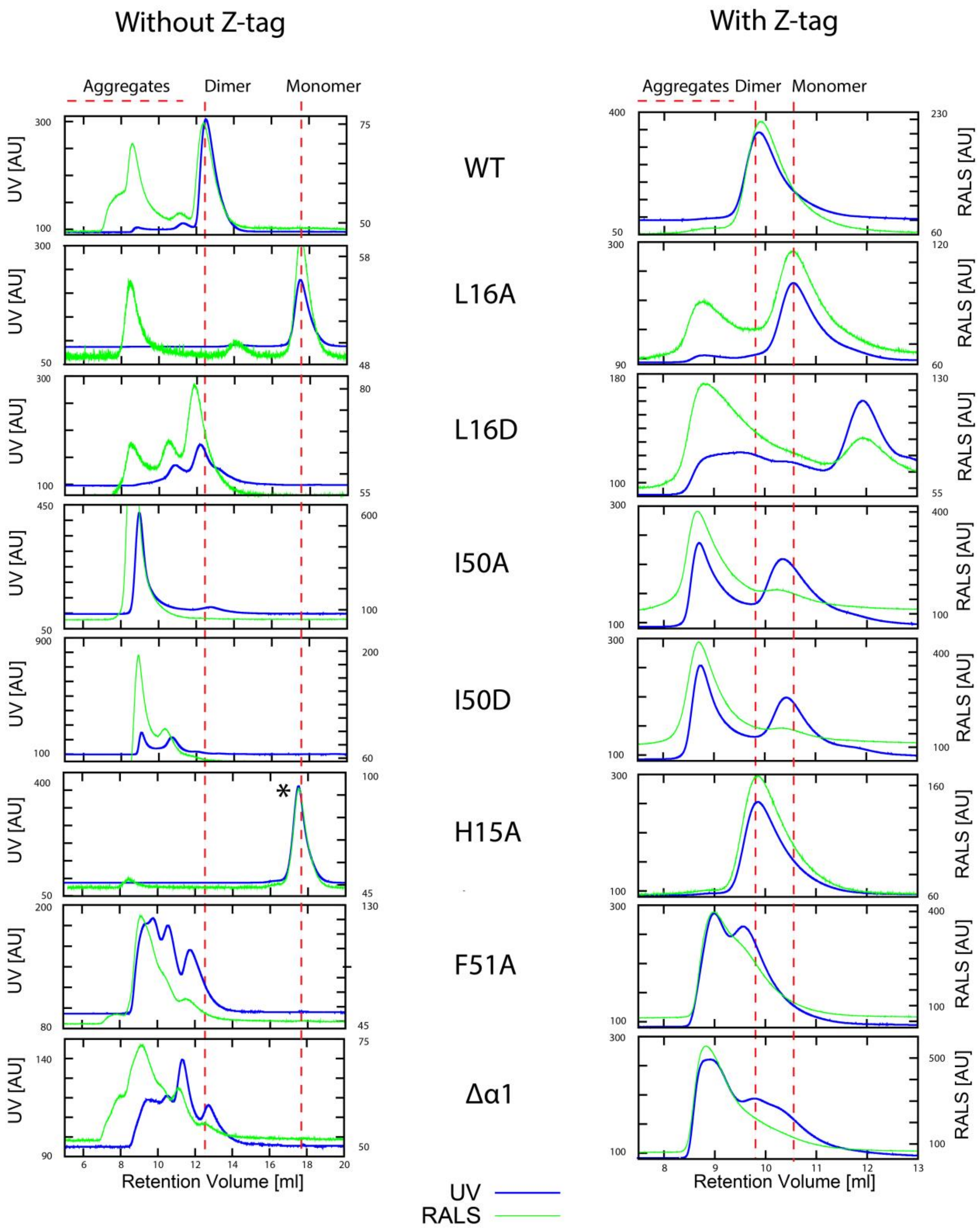

Figure C. Combined size exclusion (SEC) and static light scattering (SLS) data of wild-type and mutant END domains, without (left column) and with a solubilizing Z-tag (right

column). The plots show the elution profiles of an analytical size-exclusion column as detected by two different detectors: UV (blue line), and right angle light scattering (RALS, green line). The scales to the left and right of each plot show arbitrary units of UV and RALS, respectively. See Experimental methods for technical details. The dashed red line indicates retention volumes of different species (monomer, dimer and aggregated states) and corresponding states are indicated on top of each line. Elution peaks marked with an asterisk (H15A) indicate differences found for apparent molecular weights based on retention times and calculated from refractive index (RI) and RALS data. RALS peaks without a considerable UV peak are usually an artifact of very large aggregates and therefore neglected in the analysis (e.g. L16A, w/o Z-tag).

Figure D

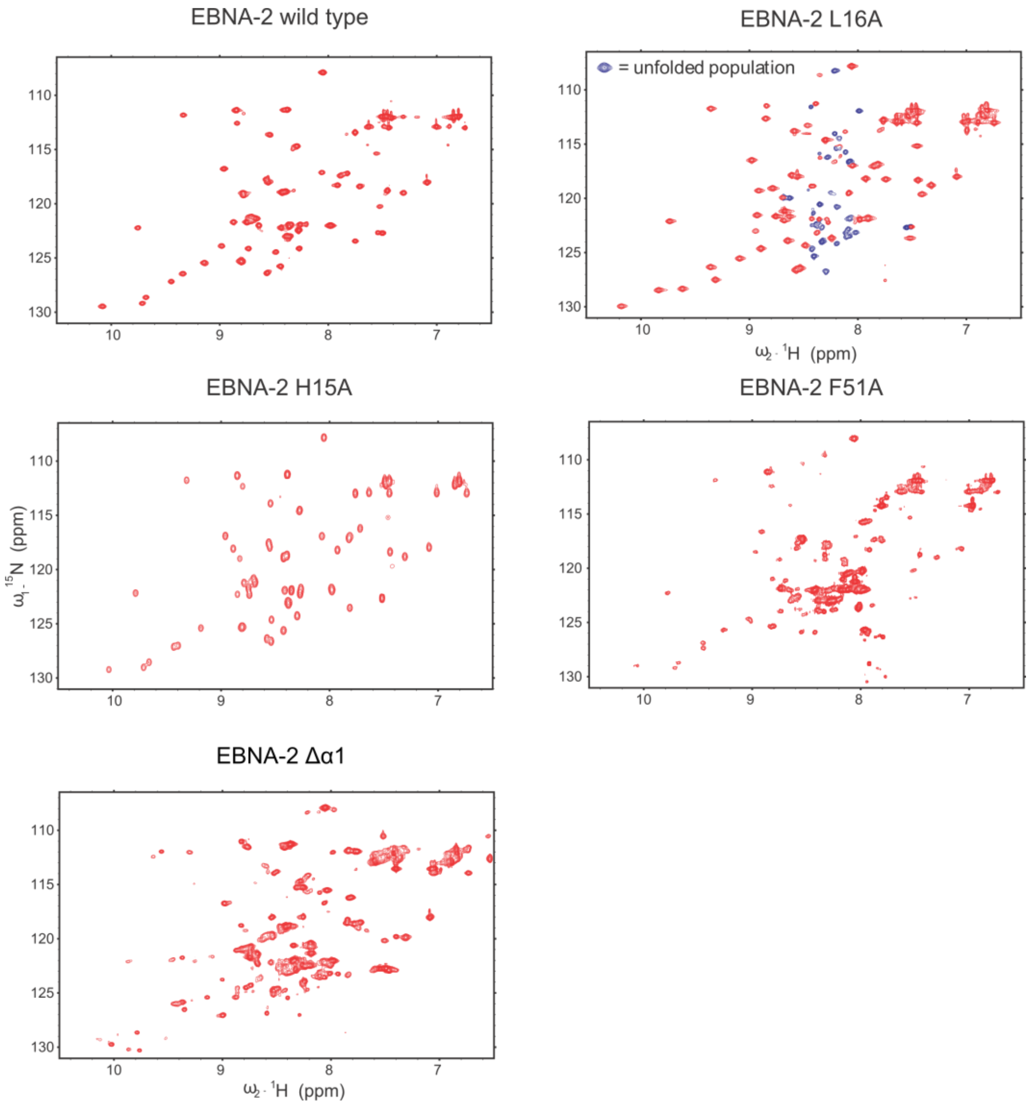

Figure D. Comparison of $^{1}H,^{15}N$-HSQC spectra of the wild-type and L16A, F51A, H15A, and Δα1 mutants of the END domain. As evident from the dispersed peak positions, these mutants are folded. The increased number of peaks in the spectrum of mutant L16A confirms the presence of an unfolded monomeric population (see also SLS data, Fig. S3). The monomer signals of L16A (blue) exhibit chemical shifts between 8-8.5 ppm, which is indicative of an unfolded protein. In conclusion, the data suggest that the L16A mutant is in equilibrium between a folded dimeric state and an unfolded monomeric state. At higher concentrations (NMR concentrations of 1 mM), this equilibrium shifts towards the folded dimer, compared to the lower concentrations of 160 µM used in SLS experiments. The spectra of the F51A and the Δα1 mutants display signals corresponding to a dimeric END domain, but additional signals suffering from line broadening suggest the presence of sample aggregation. This is consistent with the results for those mutants by SLS (Fig. S3).

Supporting Material

Figure E

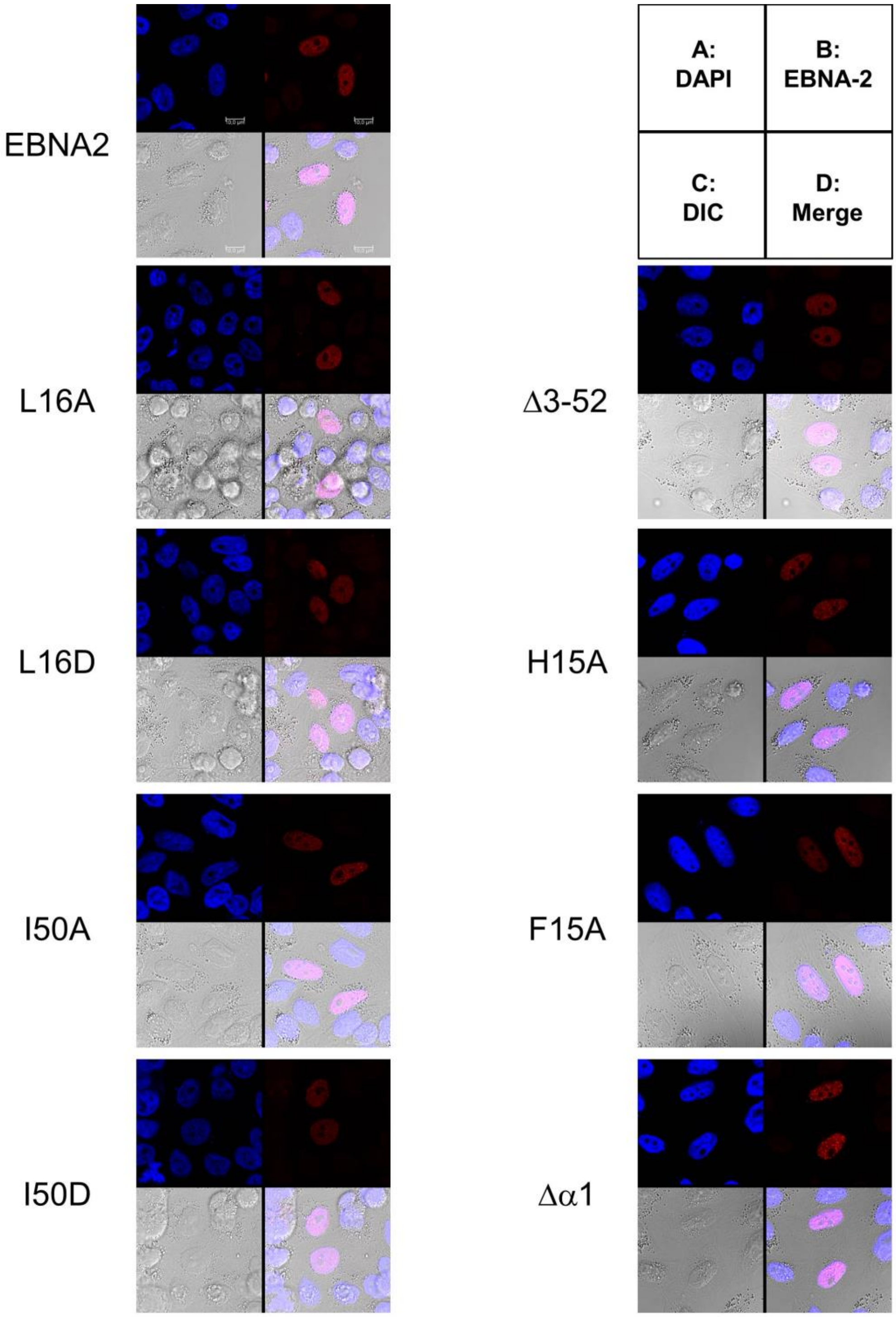

Figure E. Subcellular localization of EBNA-2 and EBNA-2 mutants. Hela cells were transfected with 1.5 µg of EBNA-2 expression constructs followed by staining with anti-rat-anti-EBNA-2 antibody plus anti-rat-Cy3 and nuclear staining with DAPI. Images were taken with a confocal laser scanning microscope TCS SP5, 63/1.4xOil objective, zoom 3.6 A Single optical plane is shown. (A) DAPI staining (B) EBNA-2 staining (C) Digital interference contrast (DIC), (D) merge of DAPI, EBNA-2 and DIC. Scale bar: 10 µm.

Figure F

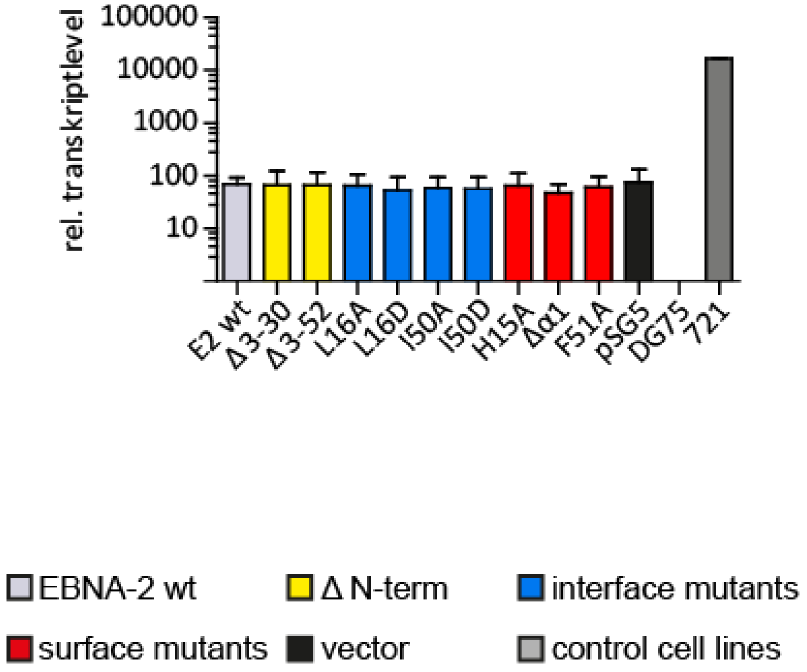

Figure F. Neither EBNA-2 nor EBNA-2 mutants activate the endogenous viral C promoter in Eli-BL cells. $1x10^7$ Eli-BL cells were transfected with expression constructs for EBNA-2, N-terminal deletion mutants, END domain mutants or the corresponding control vectors (pSG5). Relative transcript levels originating from the viral C promoter were determined by real-time RT-PCR. All transcript levels were normalized to actin. EBV negative DG75 and EBV positive 721 cells were included as controls. The results of three independent experiments are shown. Error bars indicate the standard deviations.
